# Preference for natural borders in rice paddies by two treefrog species

**DOI:** 10.1080/19768354.2018.1475301

**Published:** 2018-06-01

**Authors:** Jordy Groffen, Amaël Borzée, Yikweon Jang

**Affiliations:** Department of Life Sciences and Division of EcoScience, Ewha Womans University, Seoul, Republic of Korea

**Keywords:** *Dryophytes japonicus*, *Dryophytes suweonensis*, hylids, modernisation, conservation, amphibians, threats

## Abstract

In the Republic of Korea, one of the biggest threats to amphibians is habitat modification such as urbanisation and land conversion. With the loss of natural habitats, rice paddies play an important role as substitute habitats for amphibians that originally inhabited wetlands. However, since the 70’s, traditional rice agriculture has been modernised, leading to an increase in the number of concrete ditches and roads bordering rice paddies. This modernisation could have affected the distribution and density of amphibians. In this study, we investigated the preferred position, based on the advertisement calls for two treefrog species (*Dryophytes japonicus* and *D. suweonensis*), in relation to different types of borders such as natural ditch, concrete ditch, one-lane dirt road and vegetation. The results show that treefrogs seem to avoid rice paddies with concrete ditches, and with no ditch, which provided no resting microhabitat. The sides of the paddies preferred by the two treefrog species were the ones with vegetation of 30 cm wider or higher, while the two species seemed to avoid the side of paddies with roads. Our results are important for the conservation of anuran species in rice paddies in general as it highlights the need for vegetated areas, preferentially along natural ditches.

## Introduction

In fast developing countries, including the Republic of Korea (hereafter Korea), one of the largest threats to biodiversity is habitat modification, such as urbanisation and land conversion. Because of urbanisation, natural habitats are replaced by urban infrastructure such as houses, buildings, roads and other impermeable surfaces (McDonnell et al. 1993).

More than 7000 years of rice cultivation in Asia have resulted in wetland organisms adjusting their life cycle to the seasonal variation associated with rice farming (Fuller et al. 2007). Some species have adequately adapted to exploit this relatively new ecosystem and have subsequently flourished (Moriyama 1997). Since the 1970s, however, the modernisation of agriculture (Ho 1982; Park et al. 2005) has changed rice paddies (synonym for rice fields) ecosystem dramatically (Kiritani 2000). In this modernised system in Korea, often the water is pumped into paddy fields via pipes and is drained from fields into deep, U-shaped, concrete ditches in which the water level may be ≥70 cm below the rice paddies. The difference in water level between the rice paddy and modern drainage channels is in many cases too large for some aquatic organisms, such as for *Rana porosa,* to move between them (Fujioka and Lane, 1997; Saitoh et al. 1998; Katano et al. 2003). The changes in water management have led to habitat fragmentation and degradation (Fahrig et al. 1995) and has caused amphibian declines in some areas of the world (Verrell 1987; Blaustein and Wake 1990). For instance, frog populations decreased because of changes in rice fields’ environments in Japan (Moriyama 1997).

After the Korean War (1950–1953), urbanisation in Korea increased about five-fold, resulting in strong economic growth (Choi et al. 2006). Following the loss of natural habitats, rice paddies started playing an important role as substitute habitat for organisms that originally inhabited wetlands (Moriyama 1997; Lawler 2001). Rice paddies are seasonally exposed to drying and flooding, and as a result they are viewed as semi-natural wetlands with hydroperiods matching with anuran breeding requirements (Heckman 1979; Hidaka 1998).

In Korea, rice paddies are typically clustered together for ease of irrigation and management. Thus, a rice-paddy complex refers to a contiguous assemblage of paddies, which are divided by narrow banks or levees. Rice paddies are irrigated and connected to various habitats, such as grasslands, forests, and irrigation ponds, through irrigation systems. An increasing number of rice paddies are transformed to this new style of rice paddies, and they are consequently surrounded by roads and concrete ditches. In this study we investigated the preferred locations of two closely-related treefrog species, *Dryophytes japonicus* and *D. suweonensis*, within rice paddies, in relation to five different types of rice paddy borders: natural ditch, concrete ditch, no ditch, one-lane dirt road and vegetation. We hypothesised that both species would be found closer to natural ditches and vegetation, than to other habitat borders, due to the first two providing more grass and other plants, which are preferred for shelter when resting by both species (Borzée et al. 2016a). The preferred border is important for the conservation of anuran species in rice paddies.

## Material and methods

### Study species

Observations for rice paddy border preference were conducted for two treefrog species: the Japanese treefrog (*Dryophytes japonicus*) and the Suweon treefrog (*D. suweonensis*). These two species were previously allocated to the *Hyla* genus (Duellman et al. 2016). While *D. japonicus* is widespread throughout the North East Asia, *D. suweonensis* is sympatric with *D. japonicus* on the western coastal plains of Korea. Due to recent population declines, the status of *D. suweonensis* is classified as ‘endangered’ by the Korean government (Ministry of Environment of the Republic of Korea 2012) and the International Union for Conservation of Nature (IUCN 2017). Distinguishing these two species based on morphology is difficult (Borzée et al. 2013). However, their advertisement calls are species specific (Jang et al. 2011; Park et al. 2013), amenable to unaided acoustic monitoring. Both treefrog species are mainly found in rice paddies, as natural wetlands adequate for breeding are extremely rare (Borzée and Jang 2015). Both species spawn in shallow water, *D. japonicus* between late April and early July, while *D. suweonensis* breeds from May to late June.

### Calling location within rice paddies

The study areas were in the city of Paju, Gyeonggi province, in the Republic of Korea. Field work was conducted between 17 June and 1 July 2013 in rice paddies (Table 1), between 19:00 and 02:30 the next day, matching the peak calling activity of *D. japonicus* during breeding season (Borzée et al. 2016b). During the sampling period, the rice paddies were flooded, there was no rain and the temperature remained consistently warm, typical of weather conditions for the breeding activities of the species.

**Table 1.** The locations of the 16 rice paddies surveyed for the positioning of the treefrogs in relation to different habitat borders. N indicates the number of treefrogs per species (*Dj* for *Dryophytes japonicus*, and *Ds* for *Dryophytes suweonensis*)*.* The presence (1) or absence (0) of different type of habitat border in the particular rice paddy; vegetation (Veg.), natural ditch (Nat. ditch), concrete ditch (Con. ditch), no ditch or one-lane road (Road). Multiple habitat borders could be present in one rice paddy.

Location			*N*		Present (1) or absent (0) habitat border				
Site	Latitude	Longitude	*Dj*	*Ds*	Veg.	Nat. ditch	Con. ditch	No ditch	Road
1	37.887.837	126.738.795	7	1	1	1	0	0	1
2	37.888.029	126.740.352	8	0	1	0	1	0	1
3	37.888.426	126.740.644	4	1	1	1	0	0	1
4	37.888.174	126.744.492	22	0	1	0	0	1	1
5	37.885.761	126.741.526	10	0	1	1	0	0	0
6	37.882.652	126.746.200	10	2	1	1	0	0	0
7	37.886.750	126.748.209	4	1	1	0	1	0	1
8	37.892.336	126.751.129	5	1	1	1	0	0	1
9	37.893.108	126.750.914	0	2	1	1	0	0	1
10	37.903.413	126.766.134	13	0	1	0	0	1	1
11	37.904.197	126.766.806	4	0	1	0	0	1	0
12	37.902.051	126.770.050	1	0	1	0	1	0	1
13	37.901.636	126.769.506	9	2	1	1	1	0	1
14	37.747.418	126.759.736	9	0	1	0	0	1	1
15	37.752.597	126.756.610	0	2	1	0	0	1	1
16	37.899.175	126.766.500	17	3	1	1	0	0	1
Total			123	15	16	8	4	5	13

**Figure 1.**
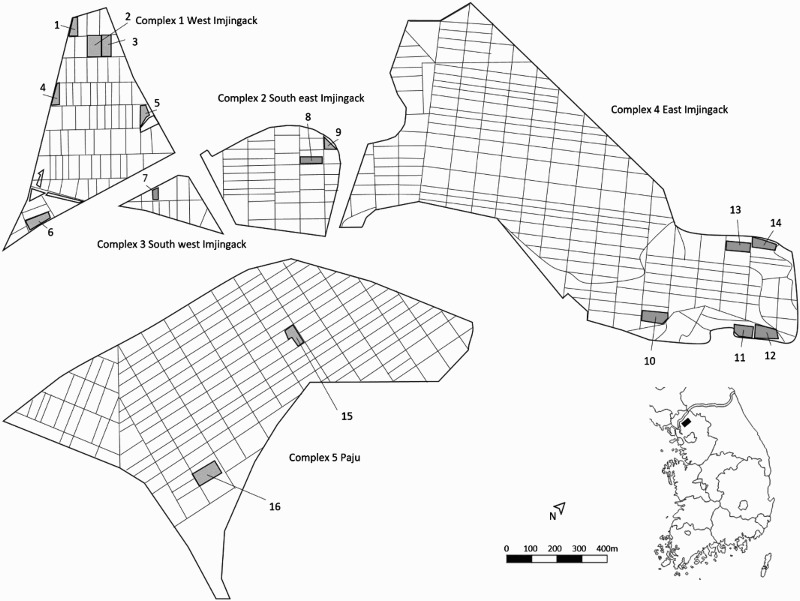
Map of the 16 rice paddies (grey) within the five different rice complexes in the city of Paju, province of Gyeonggi, in the Republic of Korea. Rice paddies next to each other were not observed on the same night. Rice complex 5 was located approximately 15 km south of the other complexes.

Data for this study were collected alongside data published in Borzée et al. (2016b). Sixteen rice paddies were randomly sampled in five different rice-paddy complexes (Figure 1). Each paddy in a complex was given a unique number between 1 and 16, and rice paddies were randomly chosen for acoustic monitoring (Brandao Apps 2010). The minimum distance between adjacent rice paddies was 85 m, and rice paddies less than 100 m apart were not sampled on the same night to avoid the possibility of sampling the same individuals. In the study, rice-paddy complexes were separated from each other either by more than 2000 m or by non-crossable landscape elements (Pellet et al. 2006) and were therefore considered as independent for statistical analyses (Borzée et al. 2016b).

After a rice paddy was selected, LED markers were placed on two adjacent banks of the rice paddy every three or five metres, in numbers low enough not to disturb the production of advertisement calls. Following the setting of these markers, a Sony PCM D-50 recorder, fitted with a directional microphone with a windshield was used to record along two continuous banks, by directing the microphone to the opposite bank. In addition, two people recorded on prepared maps of the rice paddies the location where the treefrogs were calling. Each person was given a separate side of the rice paddy, and the distance from the bank to the location of the calling individual was assessed by using the LED markers. Acoustic monitoring was conducted in a five-min period, with minor variations due to the time needed to walk around the rice paddies. This protocol was conducted only once per rice paddy. The results obtained from the two maps and the recordings were compared, and only matching data were kept for further analysis. For a more detailed description of methods and equipment used in the current study see Borzée et al. (2016b).

The physical characteristics of all four sides of the rice paddies were noted: vegetation on the banks of a rice paddy, one-lane road adjoining a rice paddy and the kind of ditch. If a border had higher and wider vegetation than 30 cm, is was categorised as ‘vegetation’. A one-lane road was defined as a paved road used for agriculture, with infrequent automobile use. Three kinds of a ditch were noted: natural, concrete or absent. Ditches were used for irrigation, and concrete ditches had a rectangular cross section and were free of any vegetation whereas natural earth ditches were vegetated, regardless of the steepness of their banks. For the no-ditch borders, no ditch was present, but a small mud bank was present to separate the two rice paddies, for agricultural purposes. These mud banks were not vegetated during the study period.

#### Statistical analyses

None of the variables (road present/absent, vegetation present/absent, type of ditch present/absent) was correlated with each other (*R* ≤ 0.16, *P* ≥ 0.059). Besides, none of these variables was correlated with frogs’ individual ID, and we therefore subsequently consider each individual to be independent of each other (Frog ID and Pearson’s Correlation test with: vegetation: *R* = 0.13, *p* = 0.133; road: *R* = 0.19, *p *= 0.230; ditch: *R *= −0.19, *p *= 0.210). Road denoted the absence (0) or presence (1) of a one-lane road, and ditch was divided into no ditch (0), natural ditch (1) or concrete ditch (2). Multiple borders were present in a rice paddy, varying between three and five. Rice paddies displayed different type of borders, and each type was treated independently in the analyses.

To assess the impact of roads, ditches, and vegetation, each rice paddy was divided into two equal parts, using the central line parallel to the bank of the rice paddy. Vegetation was included in the analysis as the absence (0) or presence (1) of plants. The location of each treefrog was noted between the two halves, in relation to these three features. ‘0’ was recorded when the frog was on the bank or less than a metre away from the bank.

For the first analyses, the two treefrog species were pooled together. Three types of statistical tests were used to discriminate preferences for the different landscape features. We used Fisher’s exact test to discriminate for the preference for sides of rice paddies with one-lane road or without road, set as nominal variables, the null hypothesis being no difference in frog numbers between ‘with road’ and ‘without road’. We used binomial test to discriminate between the presence of treefrogs on halves of paddies with and without vegetation, and the closest and furthest from the one-lane roads. In both cases there were no differences in the number of frogs present between the two halves. Finally, we used chi-square tests for differences in the number of frogs calling from rice paddies, and paddy-halves bordered by the three types of ditches, as well as to test variations in distance to the bank in relation to the types of rice paddy borders and frog species. All statistical analyses were computed using SPSS v21.0 (SPSS, Inc., Chicago, USA).

## Results

### Presence in rice paddies

**Figure 2.**
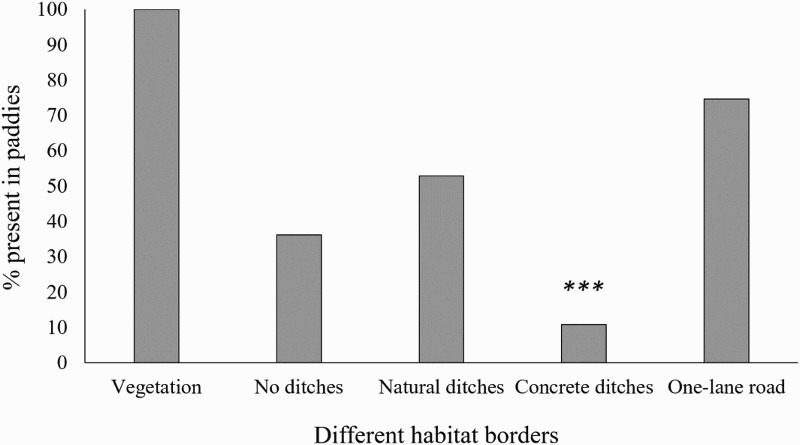
The grey boxes indicate the percentage of frogs when present in the rice paddies with a certain habitat border. *** significant less than other borders, *P* < 0.001.

During the study period, 138 treefrogs (*D. suweonensis, n* = 15 and *D. japonicus, n* = 123) were recorded in 16 different rice paddies. All rice paddies where treefrogs were recorded had at least one side of the rice paddy bordered with vegetation (100%, *n* = 138; when analysed independently for bordering features). Treefrogs were more often observed in rice paddies bordered by one-lane roads instead of those with no road (74.64% of observed frogs, *n* = 103; Fisher’s exact test, *p* = 0.008). Both treefrog species were more commonly found in rice paddies with natural ditches (52.90%, *n* = 73) and paddies without ditches (36.23%, *n* = 50), compared to paddies with concrete ditches (10.87%, *n* = 15; Chi-square test; *χ*^2^ = 37.09, *df* = 2, *p* < 0.001; Figure 2).

### Preferred border of the rice paddy

**Figure 3.**
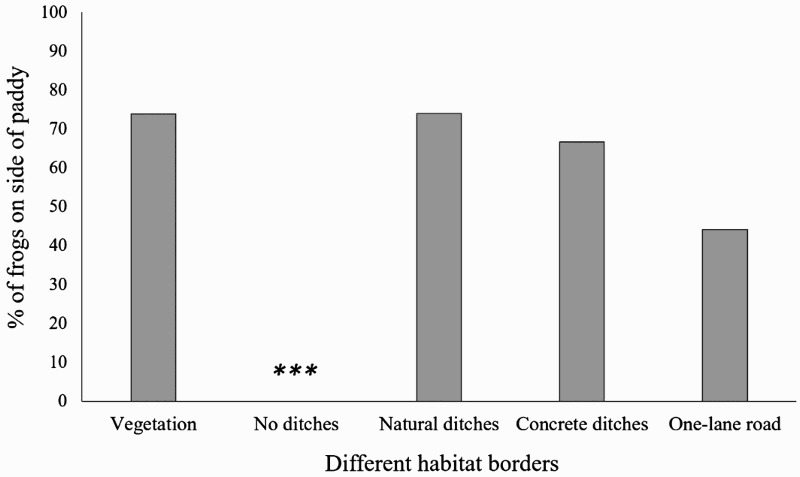
The grey boxes indicate the percentage of frogs recorded on a side of a certain habitat border. *** significant less than other sides, *P* < 0.001.

Both species were found nearest to the vegetation border of rice paddies (73.91%, *n* = 101; Binomial test, *p* < 0.001), compared to locations near other border-types*.* Frogs within a rice paddy preferred the side furthest from the road (55.79%, *n* = 77; Binomial test, *p* < 0.001), favouring the side with a natural ditch (73.97%, *n* = 54) or a concrete ditch (66.66%, *n* = 10) over no ditch (*n* = 0; Chi-square test, *χ*^2^ = 68.08, *df* = 2, *p* < 0.001; Figure 3).

### Distance from bank

The type of rice paddy borders did not affect the distance of the frogs from the rice paddy banks (Chi-square test, *χ*^2^ = 51.77, *df *= 5, *p* < 0.5). However, there was a difference in distance from bank between the two frog species: *D. japonicus* was found closer to all types of rice paddy borders compared to *D. suweonensis* (Table 2; Chi-square test, *p* < 0.001).

**Table 2.** The mean distance (in meters ± SEM) to the closest border (vegetation, natural ditch, concrete ditch or road) of the two treefrog species is given.

Species	Mean distance closest border (m ± SEM)							*n*
	Vegetation	*n*	Natural ditch	*n*	Concrete ditch	*n*	Road	
*D. suweonensis*	12.6 ± 2.06	12	12.9 ± 2.27	10	20.0 ± 0.00	1	14.4 ± 3.17	7
*D. japonicus*	1.58 ± 0.24	89	2.39 ± 0.38	44	1.11 ± 0.73	9	1.67 ± 0036	45
Both species	2.84 ± 0.47	101	4.33 ± 0.76	54	3.00 ± 2.00	10	3.35 ± 0.78	52

## Discussion

In all cases, treefrogs were found in rice paddies with at least one border with vegetation >30 cm high or wide, which was also the preferred side in most cases. Possibly because both treefrog species spend the day on rice paddy banks resting, hiding in grass, bush or buried underground (Kim et al. 2014; Borzée et al. 2016a), the vegetation border is the best habitat. While both species were often present in rice paddies with natural ditches or no ditches, the natural ditch was preferred as no treefrog were found on the sides without a ditch. Natural ditches often have more vegetation, thus providing a more suitable habitat for both species (Borzée et al. 2016a).

It is suggested that the spatial separation between calling males *D. suweonensis* and *D. japonicus* is probably due to competition for calling locations (Borzée et al. 2016b). There were no differences in rice paddy or border preference between the two treefrog species. The only inter-species difference found was the distance to the bank (*D. japonicus* closer to all types compared to *D. suweonensis*), which is in line with the results of Borzée et al. (2016b). Based on the results, we can conclude that the type and characteristics of rice paddy borders do not affect the competition for calling locations between the two species.

Both treefrog species seemed to avoid rice paddies with concrete ditches, and unlike natural ditches, concrete ditches typically remain dry, which limits grass growth. However, contradicting results were found in Japan (Naito et al. 2012), in which ditches did not have an effect on the temporal or spatial distribution of anuran species, including *D. japonicus*. The study by Naito et al. (2012) examined the presence of treefrogs in rice paddies, without examining which side of the rice paddies attracted treefrogs. Typically, only one side of a rice paddy is bordered by a concrete ditch for irrigation purposes, so frogs can remain close to the sides of rice paddies without concrete ditches. One-lane roads are common near almost every rice paddy, as they have to be accessible for mowing and harvesting, so these types of borders are almost unavoidable by the frogs. Nevertheless, the treefrogs seemed to prefer the sides furthest away from the road.

Both treefrog species seemed to prefer the sides of rice paddies with vegetation or natural ditches. This finding is valuable for the development of conservation policies of anuran species, and especially for species with the same requirements as the two Dryophytes species studied here. In Korea, 11 out of 13 anuran species are primarily found in rice paddies, and three of those 11 species are listed as endangered by the National Institute of Biological Resources of the Republic of Korea (2014): *D. suweonensis, Kaloula borealis*, and *Pelophylax plancyi*. The transformation of natural ditches to concrete ditches and the development of paved roads are bound to negatively affect treefrog species, especially the endangered D. suweonensis. Besides the transformation of agricultural ditches, other agricultural changes such as the transformation of rice paddies into green houses, ginseng farms or use of the land for various other purposes, are a threat to the survival of anuran species relying on rice paddies for breeding. The agricultural changes in combination with interspecific competition for resources (Roh et al. 2014; Borzée et al. 2016b), the potential hybridisation (Kuramoto 1984), and invasive species and diseases (Borzée et al. 2017) could be fatal to the species and conservation actions such as suggested by Borzée et al. (2015) are needed.
